# Non-suicidal self-injury and its relation to suicide through acquired capability: investigating this causal mechanism in a mainly late-diagnosed autistic sample

**DOI:** 10.1186/s13229-022-00522-5

**Published:** 2022-11-12

**Authors:** Rachel L. Moseley, Nicola J. Gregory, Paula Smith, Carrie Allison, Sarah Cassidy, Simon Baron-Cohen

**Affiliations:** 1 Department of Psychology, Bournemouth University, Talbot Campus, Fern Barrow, Poole, Dorset, BH12 5BB UK; 2 Autism Research Centre, Department of Psychiatry, University of Cambridge, Cambridge, UK; 3 School of Psychology, University of Nottingham, Nottingham, UK

**Keywords:** NSSI, Suicide, Acquired capability

## Abstract

**Background:**

Non-suicidal self-injury (NSSI) has been linked with a higher risk of suicide attempts in autistic and non-autistic people. In the general population, NSSI may confer acquired capability for suicide by eroding one’s fear and avoidance of pain and death. The present study aimed to explore acquired capability as the mediator of increased suicide risk conferred by NSSI in autistic and non-autistic adults.

**Methods:**

Autistic and non-autistic adults (*n* = 314, *n* = 312) completed an online survey exploring lifetime suicide attempts, experience with NSSI, and acquired capability for suicide. We explored relationships between lifetime incidence of NSSI and lifetime suicide attempts via three facets of acquired capability (pain tolerance, reduced fear of death, and mental rehearsal of suicide). In self-harming participants (224 autistic and 156 non-autistic), we explored whether particular types and features of NSSI might be especially associated with capability and through that with suicide: namely engagement in scratching, cutting, and self-hitting, and engaging in more numerous forms of NSSI.

**Results:**

While a higher frequency of NSSI was associated with all three facets of acquired capability, only reduced fear of death and mental rehearsal of suicide mediated an indirect relationship with lifetime suicide attempts. NSSI also directly predicted more numerous suicide attempts. Autistic people tended towards reduced fear of death and mental rehearsal regardless of NSSI status. Among self-harming autistic and non-autistic participants, cutting and an increased number of NSSI behaviours were associated with lifetime suicide attempts directly and indirectly via acquired capability. In both groups, self-hitting was associated with lifetime suicide attempts only via acquired capability.

**Limitations:**

Our cross-sectional methodology negates inferences of directionality. While we controlled for age, our samples were poorly matched, with the autistic group two times older on average. The autistic sample, predominantly late-diagnosed, female and highly qualified, were unrepresentative of the whole autistic community.

**Conclusions:**

Our data suggest that acquired capability, as measured herein, is an incomplete explanation for the association between NSSI and suicide risk. A broader construct with stable and transient facets may offer greater explanatory power, but it is probable that other variables explain or provide additional means through which this association arises.

**Supplementary Information:**

The online version contains supplementary material available at 10.1186/s13229-022-00522-5.

## Background

Non-suicidal self-injury (NSSI) describes deliberate and directly harmful behaviours (such as cutting, scratching or burning) which inflict pain and/or damage to the body.^1^ In non-autistic people, it has been reported to serve a variety of intra- and interpersonal functions, including escaping intolerable emotional states, generating desired emotions or sensations, communicating distress, or meeting interpersonal needs [1–3]. Crucially, NSSI is differentiated from a broader taxonomy of self-injurious thoughts and behaviours by the absence of suicidal intent [4]. Despite this, a robust relationship exists between NSSI and increased likelihood of suicide ideation, attempts and deaths [5–8].

There is emerging evidence that the same link between NSSI and later suicidality exists in the autistic population, who exhibit markedly higher rates of suicide attempts and deaths [9–11]. Historically, self-injury in autistic people has been perceived as a “challenging behaviour” [12] or a manifestation of stereotypy [13–16], especially when studied in minimally verbal autistic individuals with intellectual disability. In contrast to NSSI as seen in non-autistic people, these self-injurious behaviours are stereotypic and unconcealed, occurring without clear intent of causing harm and sometimes in conjunction with externally directed aggression, and associated with low communicative ability and adaptive function [17–20]. Only very recently has research reflected that autistic adults without intellectual disabilities engage in behaviours which resemble normative patterns of NSSI in their nature and functional purpose [21–23]. Qualitative analysis suggests some autistic adults perceive NSSI as a “coping mechanism”, an escape from “intolerable anxiety”, “atomic pressure” and “being lost” [22, 23]; quantitative approaches accordingly link NSSI with alexithymia, depression and anxiety [22]. Undiscerning of intent, some studies report higher rates of suicidal and non-suicidal self-injury in autistic children, adolescents and adults [24–27], linking these with compulsivity and impulsivity, insistence on sameness, hyperactivity, low mood and emotional dysregulation [28, 29].

The fact that these papers assimilated NSSI within a broader concept of “suicidal thoughts and behaviours” disallows any attempt to examine NSSI as a specific risk marker for future suicide ideation, plans, attempts and deaths. There are, to our knowledge, just two studies which did report a specific association between non-suicidal NSSI and suicidal behaviour in autistic people [30, 31]. One of these was suggestive of a particular suicide risk posed by certain NSSI behaviours (namely cutting) [31]. In the same study, where a high proportion of the autistic sample had reported neutral or positive views about their NSSI [22], there was no connection between distress associated with NSSI and suicidality.

The association between self-injury that is distinctly non-suicidal and later suicide risk is an important feature in both autistic and non-autistic populations and requires explanation. In the general population, a putative mechanism was proposed in the form of acquired capability for suicide [32, 33]: from this theoretical standpoint, exposure to physically painful and emotionally provocative events [34, 35] results in the development of pain tolerance and fearlessness about pain and death, such that individuals can enact the desire to end their own life if it occurs. Identified as a particularly potent means of accruing suicide capability [36–40], NSSI is believed to habituate individuals to pain and thus increase their tolerance while eroding their fear of it [41, 42]. Individuals who have acquired the capability for suicide via NSSI are proposed to be consequently less frightened of what is entailed in a lethal suicide attempt [36, 43]. They are also more likely to mentally rehearse suicide plans, further eroding fears of death [44].

In terms of acquiring the capability for suicide attempts, studies in non-autistic people suggest that certain features of NSSI bear particular weight. Just as a greater risk of suicide is associated with a wider variety of NSSI behaviours and with more violent and damaging methods [45–47], these features likewise predict the greatest increases in acquired capability [43, 48]. Interestingly, the type of NSSI behaviour appears more important than the *function* of that behaviour in predicting the transition from suicide ideation to attempts [49], consistent with the notion that an individual’s actions may override their intent in building suicide risk. Just as previously observed in an autistic sample [31], cutting emerges from research in the general population as particularly strongly linked with later suicide attempts; it is a particularly painful, graphic and physically damaging means of self-injury which might as such be particularly associated with building pain tolerance and fearlessness of pain and death [49–51].

Considering the previous lack of association between how autistic participants felt about their NSSI and their higher suicide risk [31], the present study aimed primarily to re-investigate the association between NSSI and suicide attempts in autistic and non-autistic adults with particular attention to acquired capability as the hypothesised mediator of this relationship. There is a paucity of literature concerning the development and significance of acquired capability in relation to suicide in autistic people, though three studies have drawn tentative links between acquired capability and suicide attempts [52–54]. One of these, an analysis addressing a different question, included the same autistic sample as the present investigation: it highlighted the potential relevance of reduced fear of death and mental rehearsal of suicide plans for suicide attempts [54]. The present study, which includes a comparison group, aimed to consequently expose differences in the strength of relationships between NSSI, acquired capability and suicide attempts. Pursuing threads of inquiry from our previous analysis [31], a secondary goal was to explore relationships between particular types/methods of NSSI, acquired capability and suicide attempts; whether risk incurred by specific forms of NSSI was associated with features such as pain inflicted and habituation; and whether a greater variety of NSSI behaviours was likewise associated with acquired capability and suicide risk via greater pain inflicted and greater habituation.

## Method

### Participants

The autistic sample (*n* = 314) was the same as that described in a previous study [54]. They were recruited via advertisement on social media, Autistica’s research network and the Cambridge Autism Research Database; we also contacted participants on our mailing lists from previous studies [22, 31]. Trusting in participants’ honesty as regards self-reporting their autism diagnosis, we did not clinically validate these diagnoses, though we did ask for details of the place and date of diagnosis. The comparison group of non-autistic adults (*n* = 312) mainly comprised students from Bournemouth University, plus some recruited from social media: asked about neurodevelopmental conditions, none of these endorsed an autism diagnosis or suspected autism. Demographic information for both groups can be seen in Table 1, though unfortunately we did not possess ethnicity data for the control group. It is likely that they were mainly Caucasian/white, given the low ethnic diversity in county Dorset.

**Table 1:** Participant demographic details and scores in study variables

	Autistic group (*n* = 314)	Non-autistic group (*n* = 312)
Average age (years)	41.9 (SD: 13.4), range 18–72	21.3 (SD: 6.4), range 18–63
Average age at diagnosis (years)	34.6 (14.8), range 2–67	
Sex		
% male|female|other	26.8|72.9|3	10.3|89.7|0
Gender identity		
% Cisgender male	25.2	10.3
% Cisgender female	57.3	88.8
% Non-binary|Transgender	14.6|2.9	3|6
Ethnicity		
% Caucasian/White	79.9,	
% Black|Mixed race	1.6|5.4	
% Other|No response	4.3|8.8	
Educational attainment		
% GCSEs or equivalent	94.9	99.4
% Bachelors degree (obtained or studying for)	70.1	89.1
% Postgraduate qualifications	35.7	1.6
Neurodevelopmental conditions		
% ADHD|ADD	17.2	2.2
% Dyslexia|Dyspraxia	8.9|8.6	7.1|1.9
% Other specific learning disabilities	6.4	1.3
Psychiatric conditions		
% Depression|Anxiety|combined depression and anxiety	7.9|9.6|39.8|	4.2|8.3|11.2
% PTSD/complex PTSD	10.2	
% Eating disorders	8.6	
% OCD|Other	8.6|9.4	
% Single psychiatric condition	18.2	
% Two psychiatric conditions	27.4	
% 3 + psychiatric conditions	23.2	
% No diagnosed psychiatric conditions	31.2	
History of NSSI		
% With lifetime experience of NSSI	72.6	51.3
Recency of NSSI (within self-harming groups)		
% Over two years ago	36.3	30.5
% Between 1 and 2 years ago	8.8	16.9
% Between 6 months and 1 year ago	6.6	15
% Between 1 and 6 months ago	13.6	15
% In the last 4 weeks	34.7	22.6
Average scores in study variables		
ACWRSS total	29.9 (SD 13.2), range 0–56	20 (SD 13.2), range 0–53
Pain tolerance	6.6 (SD 4.9), range 0–16	6.1 (SD 4.8), range 0–16
Reduced fear of death	7.5 (SD 5.2), range 0–16	4.6 (SD 4.2), range 0–15
Mental rehearsal of suicide	15.9 (SD 7.6), range 0–2	9.4 (SD 8.2)., range 0–2
Patient Health Questionnaire-9	4 13 (SD 7.2), range 0–27	4 9.8 (SD 6.6), range 0–27

Table 1 displays participant demographic features and scores in two of our experimental measures, the Acquired Capability With Rehearsal for Suicide Scale (ACWRSS) and the Patient Health Questionnaire-9; note that our two other scales, the NSSI-AT and the SITBI, do not yield total or subscale forms in the manner used here.

### Materials and procedure

Ethical permission for the study was granted by the Science and Technology Faculty panel of Bournemouth University. Data for the present analysis were collected between July 2020 and March 2021 as part of a larger online study which involved a number of additional standardised questionnaires (see [54]). We describe only the measures relevant to the present analysis.

#### The Non-Suicidal Self-Injury Assessment Tool (NSSI-AT)

The NSSI-AT [55] comprises a comprehensive assessment of NSSI, including the nature of self-injurious behaviours (i.e. the method of self-injury), their functional purpose as perceived by the participant, and the recency and frequency of behaviours. Participants were classified as having engaged in NSSI if they reported engaging in any number of listed behaviours at any point in their lifetime and if those behaviours were not *only* a means of practising or attempting suicide (as per [22, 31]). With one non-autistic participant excluded for this reason, the number of participants with a lifetime history of NSSI is shown in Table 1.

The present study used six indices from the NSSI-AT as predictors or mediators. Lifetime incidence of NSSI, range of NSSI behaviours, and habituation to NSSI were all continuous variables, and we also created three binary codes reflecting engagement in three common forms of NSSI. All participants were scored for lifetime incidence of NSSI: participants were coded 0 if they had never engaged in NSSI, 1 if they had hurt themselves just once, 2 if they had engaged in NSSI 2–3 times, 3 for 4–5 times, 4 for 6–10 times, 5 for 11–20 times, 6 for 21–50 times, and 7 for 50 times or more. The remaining variables were only relevant for individuals with a history of NSSI. In these participants, the range of NSSI behaviours was quantified by giving participants a score of 1 for every method/type of NSSI engaged in, such that participants with higher scores were those who engaged in a wider range (more types) of NSSI behaviours than individuals who consistently used one or two methods, regardless of frequency. Habituation to NSSI was indicated by responses to the Habituation and Perceived Life Interference section of the NSSI-AT, specifically to the six items measuring habituation (e.g. “I have had to intentionally hurt myself more deeply and/or in more places on my body over time to get the same effect”); higher scores indicated that participants had habituated or developed tolerance for NSSI and required more damaging/painful behaviours to achieve the desired effect. Finally, we created three binary indices affirming [1] or negating (0) engagement in scratching or pinching oneself to the point of bleeding or marking the skin (henceforth “scratching”), cutting oneself, and punching or hitting oneself to the point of bruising or bleeding (henceforth “self-hitting”), which were the three most common methods of NSSI in both groups.

Although not part of the scale, we created a seventh index which was presented with the NSSI-AT. As pain is understood to be important in the development of acquired capability [34], participants with NSSI experience were asked to think about the method they most frequently used and indicate, on a sliding scale from 1 (not painful at all) to 10 (extremely painful), how painful it usually felt.

#### Acquired Capability with Rehearsal for Suicide Scale (ACWRSS)

There is conceptual debate as to the nature of acquired capability [56] and the psychometric quality of existing measures [57]. We employed a brief screening measure based on a three-factor model of the construct [44]. The ACWRSS constitutes 7 items which load on factors pain tolerance (2 items, e.g. “I can tolerate pain much more than I used to”), reduced fear of death by suicide (2 reverse-scored items, e.g. “Even if I wanted to, killing myself is too scary to follow through with it”), and mental rehearsal of suicide plans (3 items, e.g. “I have thought of ways to kill myself that would be the least difficult for me to pull off”). Participants responded to items on an 8-point scale between “Not at all” to “Very strongly”, with higher scores in the total and subscales reflecting greater acquired capability for suicide. Though this measure has not yet received extensive use and external validation, it was seen to yield a consistent 3-factor structure across independent samples (with alpha coefficients range of 0.74–0.81), to operate invariably across males and females, to show good test–retest reliability over 2 months, and to possess strong convergent validity with items assessing suicide ideation, intent, readiness, prior suicide attempts and NSSI ideation and behaviours [44]. Internal consistency (*α*) was good for the autistic sample (total: 0.78; pain tolerance: 0.78; fear of death: 0.75; and mental rehearsal: 0.84). For the non-autistic group, internal consistency was good for the total scale (0.81) and for the pain tolerance (0.81) and mental rehearsal (0.91) subscales, but poor for the reduced fear of death by suicide subscale (0.51). The average scores for both groups are given in Table 1.

#### Self-Injurious Thoughts and Behaviours Interview, short form (SITBI)

For the outcome variable in our analyses, we used a single item from the SITBI [58]: “How many times in your lifetime have you made an actual attempt to kill yourself, in which you had at least some intent to die?”. Scores in this continuous index of lifetime suicide attempts ranged from 0 to 4, reflecting choices from “Never” (endorsed by 161 autistic and 238 non-autistic participants), “Once” (51 autistic and 34 non-autistic participants), “Twice” (28 autistic and 17 non-autistic participants), “Three or four times” (48 autistic and 12 non-autistic participants), to “Five or more times” (26 autistic and 11 non-autistic participants).

#### Patient Health Questionnaire-9 (PHQ-9)

As depression is a common if not strongly reliable antecedent of suicide attempts [33] and may be higher in individuals who engage in NSSI [22], we modelled current depressive symptomatology as a covariate in our analyses. The PHQ-9 [59] has recently been validated for use in autistic people [60]; it showed good internal consistency in our autistic (*α* = 0.90) and non-autistic (*α* = 0.90) samples, whose scores can be seen in Table 1. A clinical cut-off of 8 is recommended for the major depressive disorder [61].

### Analysis

Data were inspected for outliers (Cook’s test), linearity, autocorrelation (Durbin–Watson test), and homoscedasticity [62]. As NSSI and suicidality are somewhat exceptional phenomena, our predictor and outcome variables would not be expected to conform to the normal distribution; normality violations are generally not uncommon in psychiatry [63]. Normality is not a prerequisite for the bootstrapping method employed in our analyses, but nevertheless we visually examined the data of each group for total scores in acquired capability (ACWRSS), each ACWRSS subscale, and the PHQ-9, which did not raise concerns in terms of skewness or kurtosis. Of importance, though, was the significantly lower age of non-autistic participants and the unequal distribution of participants by sex, with women over-represented in both groups. Age and sex were thus controlled for as covariates in all analyses comparing the two groups.

Analysis followed two streams, the first exploring acquired capability as a mediator in the relationship between NSSI and suicide attempts in all 626 participants. To investigate this, we employed the PROCESS macro for SPSS [64], an ordinary least squares method with bootstrapping (5000 samples). With confidence levels set at 95%, the first analysis (Model 59) used lifetime incidence of NSSI as a continuous predictor and lifetime suicide attempts as a continuous outcome measure. As facets of acquired capability, pain tolerance, reduced fear of death and mental rehearsal were modelled as parallel mediators, with depression, age and sex as covariates. Potential moderating effects of diagnosis were examined for all pathways in the model, most particularly the direct (c’) and indirect effects of NSSI on suicide attempts. Based on our previous research where NSSI predicted 13% (Nagelkerke R2) of the variance in lifetime suicide attempts [36], we had calculated that with power set at 90% for this analysis (with its 8 variables), even a weak relationship between NSSI and suicide attempts would be expected to be evident with anything above 136 participants.

The second stream of analysis included only participants with a history of NSSI (224 autistic and 156 non-autistic participants): we aimed to explore whether certain forms of NSSI were particularly associated with suicide attempts via acquired capability, and whether this association was due to characteristics of these methods such as how painful they are and how much individuals habituate to them. The three most common forms of self-injury, in both groups, happened to be scratching, cutting, and self-hitting. Using the Lambda statistic, we confirmed the independence of these variables as binary predictors (where 1 indicated the presence of that form of NSSI). As a wider range of NSSI is indicative of more extreme behaviours and of habituation over time [46, 48], the range of NSSI was also selected as a predictor. We then performed four sequential mediation analyses (Model 6 in PROCESS), one for each predictor, wherein the effect of the predictor on lifetime suicide attempts was hypothesised to be serially mediated by pain experienced (mediator 1), which would be expected to predict habituation (mediator 2) and from this, acquired capability for suicide (mediator 3, using ACWRSS total score). In each instance, depression was modelled as a covariate. Note that because some autistic participants did not complete all items of the pain and habituation subscales of the NSSI-AT, sample numbers varied slightly (between 220 and 222 of 224 possible participants) across the four analyses.

Model 6 does not allow the presence of a moderator, so autistic and non-autistic participants could not be statistically compared in these analyses. As such, these four analyses were run for each group separately, and alpha levels were corrected to *p* < 0.0125 for each batch. The analyses for non-autistic participants, as not the focus of this paper, are summarised below with regards to any differences that arose, while presented in full in Additional file. Note that across all analyses, coefficients are unstandardised; values are rounded to two decimal places bar instances when confidence intervals were very close to 0.

## Results

### Part 1: Relationships between NSSI and suicide attempts as mediated by acquired capability

The higher lifetime incidence of NSSI was associated with all three facets of acquired capability: with higher pain tolerance (path a^1^: *b* = 0.48, *p* < 0.001, CI: 0.27, 0.70; *R*^2^ = 0.12, *F* [6, 619] = 14.13, *p* < 0.001); with reduced fear of death by suicide (path a^2^: *b* = 0.46, *p* < 0.001, CI: 0.25, 0.68; *R*^2^ = 0.17, *F* [6, 619] = 21.05, *p* < 0.001); and with more mental rehearsal of suicide plans (path a^3^: *b* = 1.29, *p* < 0.001, CI: 0.99, 1.60; *R*^2^ = 0.44, *F* [6, 619] = 81.66, *p* < 0.001). The diagnosis did not moderate relationships between NSSI incidence and pain tolerance or reduced fear of death but was directly predictive of reduced fear of death by suicide (*b* = 1.55, *p* = 0.0299, CI 0.15, 2.95). For the mental rehearsal aspect of acquired capability, diagnosis exerts both a main effect (*b* = 0.37, *p* < 0.001, CI 4.40, 8.34) and a moderating effect on its relationship with NSSI incidence (path ^3^: *b* = -0.82, *p* < 0.001, CI -1.20, -0.44). Respectively, these reflected that autistic status was associated with higher mental rehearsal of suicide plans and that the relationship between higher frequency of NSSI and greater mental rehearsal was stronger in non-autistic (*b* = 1.29, *p* < 0.001, CI 0.99, 1.60) than in autistic participants (*b* = 0.47, *p* < 0.001, CI 0.21, 0.73). These effects were independent of depression (positively associated with each facet of acquired capability), age (positively associated with reduced fear of death), and sex (see Additional file 1).

**Fig. 1:**
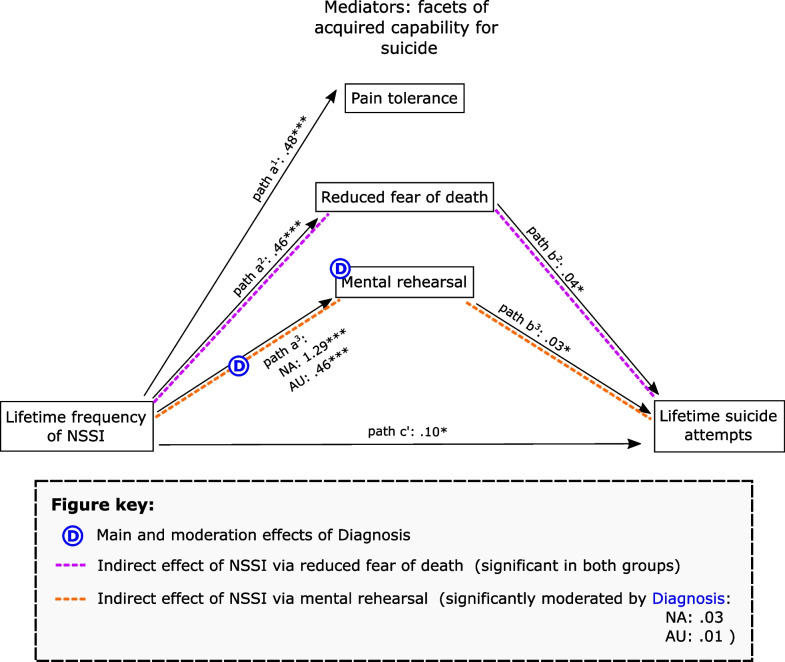
Direct and indirect relationships between lifetime incidence of NSSI and lifetime suicide attempts. Lines in black reflect relationships significant in both groups: coefficients are marked with a single asterisk where significant at a corrected threshold of *p* < .025, and with three asterisks where significant at *p* < .001. Effects of Diagnosis are depicted in blue where they affect variables (main effects) and relationships (moderation effects); where this occurs, coefficients are displayed for both groups. Dotted lines in purple and orange reflect the indirect effects of NSSI on lifetime suicide attempts through mediating variables

The model predicting lifetime suicide attempts (*R*^2^ = 0.34, *F* [12, 613] = 26.63, *p* < 0.001) was contributed to by higher lifetime incidence of NSSI (path c’, which was significant in both groups [*b* = 0.10, *p* = 0.0012, CI 0.04, 0.15]), reduced fear of death (path b^2^: *b* = 0.04, *p* = 0.0072, CI 0.01, 0.07), mental rehearsal of suicide (path b^3^: *b* = 0.03, *p* = 0.0076, CI 0.01, 0.05), and depression (*b* = 0.02, *p* = 0.0049, CI 0.01, 0.03). While greater lifetime incidence of NSSI predicted higher lifetime suicide attempts independent of the mediators (i.e. directly), it also exerts two indirect effects. In the first, a higher frequency of NSSI predicted higher suicide attempts via reduced fear of death by suicide, an effect significant in both autistic (*b* = 0.01, bootstrapped CI 0.0007, 0.03) and non-autistic participants (*b* = 0.02, bootCI 0.01, 0.03). Higher frequency of NSSI also predicted suicide attempts via higher mental rehearsal of suicide plans; while this was significant for both autistic (*b* = 0.01, bootCI 0.0007, 0.02) and non-autistic participants (*b* = 0.03, bootCI 0.02, 0.05), a significant index of moderated mediation (*b* = -0.02, bootCI -0.05, -0.00) showed that the indirect effect of NSSI frequency on suicide attempts via mental rehearsal was stronger in non-autistic participants (Fig. 1).

### Part 2: Types and features of NSSI as predictors of acquired capability and suicidality

#### Scratching

For autistic participants, scratching was not significantly associated with any of the three sequential mediators in the model. While average pain during NSSI (the first mediator) did not predict habituation (second mediator) or indeed acquired capability (the third mediator), the expected relationship between habituation and acquired capability was observed (*b* = 0.83, *p* = 0.0032, CI 0.28, 1.39). Of additional interest was the effect of depression as a covariate which predicted greater endorsement of habituation to NSSI (*b* = 0.18, *p* < 0.001, CI 0.12, 0.23) and higher acquired capability (*b* = 0.51, *p* < 0.001, CI 0.27, 0.75). Only acquired capability, however, significantly predicted lifetime suicide attempts (*b* = 0.04, *p* < 0.001, CI 0.02, 0.05; *R*^2^ = 0.14, *F* [5, 214] = 7.22, *p* < 0.001), with no direct or indirect effects of scratching. The data for non-autistic participants showed exactly the same picture (see Additional file 1).

#### Cutting

**Fig. 2:**
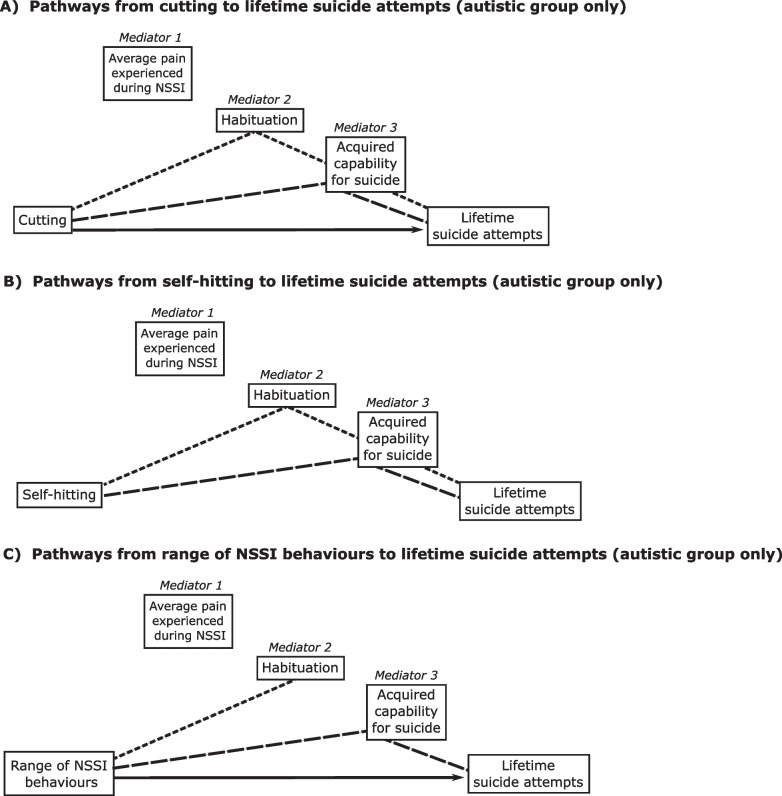
**A**–**C** Pathways between lifetime suicide attempts (outcome) and cutting, self-hitting and range of NSSI behaviours, respectively (independent predictors). Only results from the autistic group are depicted here

Though cutting was not associated with greater pain experienced during NSSI for autistic participants, the association between cutting and habituation (*b* = 0.98, *p* = 0.0131, CI 0.21, 1.75) was at the borderline of our adjusted alpha value (*p* < 0.0125). Endorsement of cutting was associated with acquired capability (*b* = 5.68, *p* = 0.0005, CI 2.52, 8.84). Interestingly, the model predicting lifetime suicide attempts was contributed to not only by acquired capability (*b* = 0.03,* p* = 0.0006, CI 0.01, 0.04; *R*^2^ = 0.24, *F* [5, 215] = 13.48,* p* < 0.001) but by cutting as a direct effect (*b* = 0.96, *p* < 0.001, CI 0.60, 1.33), and explained considerably more of the variance in lifetime suicide attempts as compared to the preceding model with scratching as a predictor. In addition to predicting lifetime suicide attempts directly, cutting exert one (albeit very weak) indirect effect on lifetime suicide attempts via habituation and then acquired capability sequentially (*b* = 0.02, bootCI 0.001, 0.04), and a stronger one via acquired capability alone (*b* = 0.15, bootCI 0.04, 0.30): these pathways are summarised in Fig. 2 part A. For non-autistic participants, cutting was associated with lifetime suicide attempts directly and indirectly via habituation and then acquired capability sequentially (see Additional file 1).

#### Self-hitting

The last specific form of NSSI, endorsement of self-hitting, was associated with habituation (*b* = 1.07, *p* = 0.0059, CI 0.31, 1.82) and acquired capability (*b* = 4.77, *p* = 0.0031, CI 1.62, 7.92) in autistic participants. It was not directly associated with lifetime suicide attempts but did exert two indirect effects: one through habituation and then acquired capability sequentially (*b* = 0.03, bootCI 0.002, 0.06), and one through acquired capability alone (*b* = 0.18, bootCI 0.05, 0.32), as depicted in Fig. 2 part B. For non-autistic participants, hitting oneself was indirectly associated with lifetime suicide attempts via the same two routes as in autistic participants (see Additional file 1).

#### Range of NSSI behaviours

Our final analyses focussed on the range or number of different NSSI behaviours participants engaged in. For autistic participants, a higher range of behaviours was associated with greater habituation (*b* = 0.31, *p* = 0.0001, CI 0.16, 0.46), higher scores in acquired capability (*b* = 1.46, *p* < 0.001, CI 0.82, 2.10), and directly predicted a higher number of suicide attempts (*b* = 0.19, *p* < 0.001, CI 0.11, 0.27). In addition to directly predicting lifetime suicide attempts, a higher range of NSSI behaviours was associated with suicide attempts indirectly via acquired capability (*b* = 0.04, bootCI 0.01, 0.07), as shown in Fig. 2 part C. Precisely, the same pattern of results was seen in non-autistic participants (see Additional file 1), alongside an additional indirect effect of a range of NSSI via habituation and acquired capability sequentially.

## Discussion

The aims of the present investigation were twofold. Primarily, we attempted to examine whether the established relationship between NSSI and suicide attempts in autistic people [30, 31] was explained by the acquired capability for suicide, and to compare the nature and strength of these relationships in autistic and non-autistic people. Secondarily, we aimed to explore whether certain forms or methods of NSSI were especially associated with acquired capability through these suicide attempts, and whether it was the features of certain methods, in this case pain inflicted and habituation, which mediated their relationship with acquired capability and with suicide attempts.

### Acquired capability as a bridge from NSSI to suicide

Since the original conception of acquired capability for suicide [32, 33], NSSI was highlighted as a particularly potent means of eroding an individual’s natural fear of pain, and thus their fear of attempting suicide. In accordance with our three-factor conceptualisation of this construct [44], relationships have been observed, in the general population, between NSSI and reduced fear of death [49], increased mental rehearsal of suicide plans [39], and greater tolerance for pain [65, 66]. As there is little research on acquired capability for suicide in autistic people, let alone its relationship with NSSI, our first analysis distilled the construct and examined its three facets as mediators of the relationship between the lifetime incidence of NSSI and lifetime suicide attempts. As per the aforementioned literature, our data corroborated relationships between higher incidence of NSSI and each facet of acquired capability. In similarity with the developers of the three-factor model [44], our two groups showed indirect effects of NSSI on more numerous lifetime suicide attempts through the mediators of mental rehearsal and reduced fear of death. While the pathways between NSSI, acquired capability, and lifetime suicide attempts appeared to operate similarly between the two groups, two moderation effects of diagnosis revealed that autistic status was associated with a weaker relationship between lifetime NSSI and mental rehearsal, and a weaker indirect pathway from NSSI to suicide attempts via mental rehearsal. We suspect that both examples of weaker behavioural contingencies reflect greater mental rehearsal and reduced fear of death in the autistic group (two main effects), *irrespective* of their engagement in NSSI. Our findings somewhat contradict those of one previous study [52], which found no group differences between autistic and non-autistic people in relation to fear of death; however, they are consistent with the greater exposure of autistic people to physically painful and emotionally provocative events across their lifespans [67–69].

Two findings stood in contrast to the traditional understanding of acquired capability [33]. The first of these was the lack of association between greater pain tolerance and lifetime suicide attempts in both groups. It is highly possible that this reflects a disconnection between our two-item self-report measure and pain tolerance in naturalistic settings, and/or between pain reported in the present day and past suicide attempts. However, this finding actually corroborates broader scepticism around the relationship between pain tolerance and suicide and the inclusion of this facet within the acquired capability construct. Pain tolerance is the least specific and reliable differentiator of suicide attempts from suicide ideation [56], with some studies even finding lower pain tolerance in individuals who had attempted suicide [70, 71]. It is possible that these findings reflect real-world nuance which is difficult or impossible to capture experimentally, as might be the case if pain tolerance influenced an individual’s choice of approach to a suicide attempt [71]. However, while the original conceptualisation of pain tolerance assumed this variable could only increase monotonically or stay stable, recent literature reflects that pain perception is influenced by numerous psychological variables [72], that it fluctuates across NSSI episodes and over time in its relation to perceived capability for suicide [70, 73]. The present study corroborates the unreliability of pain tolerance as a supposed prerequisite for suicide attempts.

A second finding contrary to the proposed role of acquired capability within the ideation-to-action trajectory [33] was the presence of a *direct* effect of lifetime NSSI on lifetime suicide attempts. While it contradicts theory, this finding is in fact again consistent with other empirical observations of direct associations between NSSI and suicide attempts in the general population [40, 43, 74]. There are, at least, two potential interpretations of this direct effect. Firstly, it may reflect the need for a more multifaceted concept of “suicide capability” [37, 75–77], which, fluctuating over time, could incorporate “baseline” capability (relatively static dispositional factors, such as genetically high pain threshold and low fear of death) as well as dynamic and situational factors (such as access to means [56, 78, 79], recent exposure to suicide attempts in others [75, 80]). Ribeiro and colleagues [81] note that although in a broad and logical sense the desire and capability to attempt suicide *are* necessary prerequisites for suicide attempts, our highly specific operationalisation of this desire and this capability do not, at present, sufficiently capture the complexity of these multitudinous variables and their interactions over time—hence why even in longitudinal investigations, suicide capability only explains a small degree of the variance in suicide attempts.

However, the second interpretation of this finding suggests we need to look further beyond acquired or broader suicide capability to understand alternative means through which NSSI might influence suicide risk. Firstly, NSSI is associated with thwarted belongingness and perceived burdensomeness [38, 82, 83], both of which the Interpersonal Theory of Suicide asserts are necessary for suicide desire/ideation [32, 33]. While NSSI is associated with poorer interpersonal problem-solving [84] and interpersonal distress [85], it has been suggested to exacerbate these states and may thus *contribute* to suicide ideation [38, 82, 83], which would be a necessary propellant for suicide capability to enable a suicide attempt. Similarly, although NSSI is associated with intolerance for psychological pain and negative affect [86–88], some suggest it may exacerbate psychological pain by precluding the development of more effective coping strategies, such that individuals are vulnerable to suicide in a scenario where NSSI fails to provide adequate escape [40, 43]. Of course, while NSSI may contribute to or exacerbate these states, an alternative interpretation lies in the possibility that these and other variables could operate as hidden factors in the relationship between NSSI and suicidality: “third” variables which, shared by both, might give rise to the appearance of a relationship between the two. For instance, in autistic populations, cognitive inflexibility [89, 90] rumination [91] and alexithymia [22, 92] have been highlighted as potential factors in NSSI and suicidal behaviour (as well as broader psychopathology), perhaps in part because they preclude adaptive means of problem-solving and emotion regulation. In the general population, psychopathology has been suggested to wholly or partially mediate links between NSSI and thwarted belongingness, perceived burdensomeness and suicide ideation [40, 82], and to explain unique variance in suicide attempts over and above contributions from thwarted belongingness, perceived burdensomeness, and acquired capability [93, 94].

It is likely, given their differences in pain perception [95–97] and lifetime experiences [67–69], that the relevance and importance of suicide capability facets differ between autistic and non-autistic populations, just as the stability and rate at which capability develops may also differ. As both mental rehearsal and reduced fear of death partially mediated relationships with lifetime suicide attempts, it remains an important research target in autistic people. However, a comprehensive exploration of links between NSSI and suicide must incorporate additional factors beyond even the broader scope of suicide capability and must accommodate the element of indeterminacy, the reality that different combinations and interactions of factors can give rise to the same behavioural outcome [81, 98].

### Specific types and features of NSSI in relation to acquired capability and suicide attempts

On the assumption that an association does indeed exist between NSSI and later suicide risk, and that this connection is partially related to suicide capability, an important question is whether different NSSI behaviours differentially create suicide capability and whether they do so in a broad sense or only in relation to certain methods of suicide [74]. Having previously observed a particular relationship between suicidality and cutting in autistic people [31], we examined this and two other common NSSI behaviours in the light of features which might mark them as particularly worrisome in terms of acquired capability and future suicidality, most notably in relation to violence, painfulness and tissue damage [33, 46, 49, 51]. Our mediation models examined the effect of each behaviour on lifetime suicide attempts as mediated by three sequential mediators: average pain experienced during NSSI, the extent to which individuals reported habituating to NSSI, and acquired capability as a whole construct. As we could not statistically compare autistic and non-autistic participants, differences between these sets of analysis may not be meaningful. We will therefore focus solely on autistic participants.

Interestingly, our binary endorsement of cutting and our continuous measure reflecting a range of NSSI behaviours behaved very similarly as variables: both were associated with habituation (albeit at trend level for cutting) and acquired capability; both directly predicted suicide attempts as well as exerting indirect effects via habituation and acquired capability sequentially, while cutting also exert an indirect effect via acquired capability alone. This is consistent with the previous literature examining these two predictors in non-autistic people. Among individual NSSI behaviours, cutting is particularly painful and provides immediate and lasting visual proof of physical damage, which is an important element of NSSI for some people [99–101]. It also straightforwardly approximates one means of suicide which could be reached by an escalation of the same behaviour, such that it is a potent means of increasing pain tolerance and reducing fear of pain and death [46, 102, 103]. Just as cutting has indeed been especially associated with acquired capability [33, 104] and with suicide attempts in the general population [49–51], so too is there a robust relationship between diversity of NSSI behaviours and suicide attempts [8, 47, 105]. Theoretically, exposure to diverse methods would be predicted to result in an increased likelihood of habituation to a range of behaviours and types of pain [46, 105]: while its association with self-reported pain tolerance is inconsistent [40], NSSI range is indeed associated with reduced fear of death [40, 43, 45, 48], and greater acquired capability as a whole [104]. In our data, the existence of a *direct* effect of both cutting and NSSI range on lifetime suicide attempts is an important indication of other variables which might explain these relationships. In non-autistic people who self-injure, both of these predictors have been linked to more severe psychopathology, greater emotion regulation difficulties, and poorer impulse control [45–47, 104, 106]; the increased versatility associated with a range of behaviours may reflect increased need, willingness and ability to engage in NSSI even when preferred means are inaccessible [46]. We cannot here determine the nature of this direct effect, but our findings are suggestive of extra risk of suicide associated with cutting and with engagement in diverse NSSI behaviours in autistic as in non-autistic people.

Interestingly, self-hitting influenced lifetime suicide attempts only via acquired capability, thus conforming most closely to original ideas around this construct [33]. Self-hitting has received less experimental attention than other forms of NSSI, in part because it tends to occur as one of a repertoire rather than as a singular NSSI behaviour [50, 104]. In non-autistic adolescents, Somer et al. [107] did identify a group that primarily engaged in self-hitting. They likened this group to latent subgroups described as “mild” or “moderate NSSI” in other samples [106, 108, 109], having a lower likelihood of psychological distress or psychopathology, lower likelihood of past suicide attempts and lower likelihood of other health risk behaviours (smoking and drinking) than groups characterised by skin-cutting or diverse means of NSSI. On the other hand, self-hitting may be more strongly associated with aggression, which has its own relationship with suicidality and acquired capability [50]. This may explain why its effect on suicidality was solely mediated by this variable.

These analyses yielded several null findings and some unanswered questions other than the aforementioned direct effect. Severe scratching and/or pinching was unrelated to any other variables: it seems likely that this reflects the broad wording of this item on the NSSI-AT, which could conceivably apply to behaviours ranging in painfulness and destructiveness. Pain experienced during NSSI was not associated with any one predictor, mediator or with suicide attempts as an outcome. While this may reflect the aforementioned inconsistency around the role of pain tolerance in relation to acquired capability and suicide risk [56], it is further notable that this index reflected pain experienced during NSSI in *general*, rather than in relation to any one of these specific behaviours. The same is true of our measure of habituation to NSSI in general—but interestingly, our analyses of cutting and self-hitting both revealed two separate indirect effects of these predictors on lifetime suicide attempts, one via acquired capability alone and one via habituation and acquired capability sequentially (albeit much weaker than the former). Interpretation of this finding can only be speculative at present, but as habituation only affected suicide attempts via acquired capability rather than independently, it is possible that this effect reflects the overlap between the two constructs: the habituation subscale of the NSSI-AT might be expected to relay most closely to pain tolerance, but less to fear of death or mental rehearsal. While these analyses are generally supportive of a pathway from NSSI to suicide via acquired capability, they corroborate our first analysis in reflecting the existence of additional mechanisms through which NSSI, and particular forms of NSSI, might influence suicidal behaviours.

### Limitations and future directions

The most prominent limitations of the present study relate to (a) the study design and operationalisation of variables, (b) the disparity between samples, and (c) their limited generalisability to wider populations. As pertains to the first of these points, our cross-sectional design was ill-equipped to test causal or directional hypotheses. While our analyses are suggestive of pathways between NSSI and lifetime suicide attempts via acquired capability, it is possible that capability was acquired *through* suicide attempts as opposed to preceding them, given the increasing ease of subsequent attempts [33, 48, 98]. Emerging views suggest that there are likely no *necessary* prerequisites for suicide but instead many possible factors and combinations of factors, distal and proximate, and that these fluctuate over time [81, 110]. Longitudinal or semi-longitudinal designs, or even computational modelling (e.g. [98, 111, 112] may afford a clearer picture of multiple possible pathways from NSSI to suicide, including any causal contributions from acquired capability.

Relatedly, our operationalisation of key variables may have been inadequate. The interpersonal theory of suicide is suggestive of a “dose–response” relationship between NSSI and acquitted capability, though this has not received appropriate experimental scrutiny. Our analyses relating to specific NSSI behaviours were limited by the use of binary variables, indicative only of the presence or absence of the behaviour but not its frequency, extent or history; nor did these binary variables reflect how other variables, like the type and intensity of pain, the presence or absence of blood, and an individual’s psychological state during NSSI, might moderate a process of acquiring suicide capability [8, 113]. Our operationalisation of pain typically experienced during NSSI, and the pain subscale of the ACWRSS, was likely inadequate. Indeed, there is presently no consensus around the optimal assessment of acquired capability in *any* population [56, 57]. The present study adopted a broader concept of acquired capability than previous investigations which focussed mainly or solely on the reduced feature of death [52, 53], but all used measurement tools designed for non-autistic samples. Quite simply, the scope and nature of suicide capability is still unknown in autistic people, as is how it may interact with other autistic features. For instance, with better operationalisation of pain tolerance, it is possible that sensory sensitivities could differentially impact the contribution of this variable to suicidality in autistic people.

The strength of our conclusions is limited by several issues concerning sampling and recruitment. Firstly, we did not clinically validate self-reported autism diagnosis or *lack* of autism diagnosis in the comparison group, relying on self-report only. Potentially greater issues lie in both the disparity between our samples and their generalisability to autistic and non-autistic populations. While we attempted to control for age and for sex in group comparisons, our groups occupied different lifespan stages: non-autistic participants as emerging adults, and autistic participants as approaching or navigating midlife. While little is known about changes in suicidal behaviour and NSSI across autistic lifespans, we know that the nature of NSSI and suicidal behaviour varies across the lives of non-autistic people [114–117]. Our efforts to statistically control for age were indubitably inadequate as a means of counteracting the different life experiences and perspectives of the groups, weakening the validity of these comparisons.

With regards generalisability, our convenience sample of non-autistic undergraduates furthermore comprises a very specific cohort unrepresentative of the non-autistic population generally [118]. Our autistic sample, too, is unrepresentative of many individuals within the autistic community. Those under- or unrepresented here include individuals with poor computer literacy; those with severe intellectual and/or communication impairments; individuals belonging to ethnic minority groups; and individuals with non-binary or transgender identities. Furthermore, a sampling bias may have been introduced if the study was more salient to those with a history of NSSI, suicide ideation or suicide attempts. Unusually for autism research, our sample was strongly skewed in favour of cisgender autistic women. The majority would be classified as “late-diagnosed”, with only 15 participants diagnosed at or below the age of 7 (a cut-off suggested in one recent study [119]). As reflected in their qualifications, they likely corresponded to a profile with fair-to-strong camouflage and compensation abilities, more normative verbal style, and possibly stronger executive function than others within the autistic spectrum [120–122]. Individuals with this profile seem disproportionately represented in studies which recruit via social media or other online methods [123, 124]. As this is the approach adopted by the present study and several others in this relatively young field [22, 30, 31, 52–54], findings are likely unrepresentative of all within the diverse autistic community. It is possible, given the differences noted between late- and early-diagnosed samples in mental health and wellbeing [125, 126], that risk and protective factors for NSSI, suicidality and psychopathology differ as a function of age at diagnosis. The topography of NSSI and its relation to psychopathology and suicide risk remains an important target for future research, which could adopt more tailored recruitment strategies for underrepresented groups (e.g. [127]).

## Conclusions

Our findings suggest that acquired capability for suicide, most notably reduced fear of death and mental rehearsal of suicide plans, partially mediates the relationship between NSSI (and specific forms of NSSI) and more numerous lifetime suicide attempts. While this finding is poignant given the higher levels of acquired capability in the autistic sample (along with weaker behavioural contingency with NSSI), the emergence of direct effects between NSSI and lifetime suicide attempts indicates that additional mechanisms underpin this association. In that relationships between NSSI, suicidality, acquired capability and other potential mediators may operate bidirectionally, longitudinal and/or computational designs may afford greater insight into stable and innate, accumulated and dynamic risk factors which could give rise to NSSI, suicidality and other deleterious outcomes in autistic people.

## Supplementary Information

**Additional file 1.** Supplementary data analysis.

## Data Availability

The data sets used and/or analysed during the current study are available from the corresponding author upon reasonable request.

## Abbreviations

ACWRSS: Acquired Capability with Rehearsal for Suicide Scale
NSSI: Non-suicidal self-injury
NSSI-AT: Non-Suicidal Self-Injury Assessment Tool, a measure of NSSI behaviours, their frequency and functionality
PHQ-9: Patient Health Questionnaire-9, a measure of depression
SITBI: Self-Injurious Thoughts and Behaviours Interview, a measure which yields an index of lifetime suicide attempts

## Footnotes

1 NSSI should not be confused with indirectly harmful/self-destructive behaviours, such as promiscuity, smoking or substance abuse. We adopt the definition as employed in DSM-5 (APA, 20,213) and by the International Society for the Study of Self-Injury (2018), wherein NSSI are directly harmful behaviours without suicidal intent. “Parasuicide”, “parasuicidal behaviours”, “self-mutilation”, “self-harm” and “deliberate self-harm” (e.g. NICE, 2013) are terms often used when the underlying motivations for self-injury are uncertain. While it is likely that non-suicidal self-injuries are encapsulated in such literature, this imprecision can obscure clarity in this field (Prinstein, 2016; Sadek, 2019), especially when trying to understand the relationship of NSSI to later suicidality.

## References

[1] Taylor PJ, Jomar K, Dhingra K, Forrester R, Shahmalak U, Dickson JM (2018). A meta-analysis of the prevalence of different functions of non-suicidal self-injury. J Affect Disord.

[2] Hepp J, Carpenter RW, Störkel LM, Schmitz SE, Schmahl C, Niedtfeld I (2020). A systematic review of daily life studies on non-suicidal self-injury based on the four-function model. Clin Psychol Rev.

[3] Peel-Wainwright KM, Hartley S, Boland A, Rocca E, Langer S, Taylor PJ (2021). The interpersonal processes of non-suicidal self-injury: A systematic review and meta-synthesis. Psychol Psychother.

[4] Nock MK, Boccagno CE, Kleiman EM, Ramirez F, Wang SB, Prinstein M, Youngstrom E, Mash E, Barkley R (2019). Suicidal and nonsuicidal self-injury. Treatment of childhood disorders.

[5] Muehlenkamp JJ, Brausch AM (2019). Protective factors do not moderate risk for past-year suicide attempts conferred by recent NSSI. J Affect Disord.

[6] Ward-Ciesielski EF, Schumacher JA, Bagge CL (2016). Relations between nonsuicidal self-injury and suicide attempt characteristics in a sample of recent suicide attempters. Crisis.

[7] Kiekens G, Hasking P, Boyes M, Claes L, Mortier P, Auerbach RP (2018). The associations between non-suicidal self-injury and first onset suicidal thoughts and behaviors. J Affect Disord.

[8] Griep SK, MacKinnon DF (2020). Does nonsuicidal self-injury predict later suicidal attempts? A review of studies. Arch Suicide Res..

[9] Kolves K, Fitzgerald C, Nordentoft M, Wood SJ, Erlangsen A (2021). Assessment of suicidal behaviors among individuals with autism spectrum disorder in Denmark. JAMA Netw Open.

[10] Kirby AV, Bakian AV, Zhang Y, Bilder DA, Keeshin BR, Coon H (2019). A 20-year study of suicide death in a statewide autism population. Autism Res.

[11] Hirvikoski T, Boman M, Chen Q, D'Onofrio BM, Mittendorfer-Rutz E, Lichtenstein P (2020). Individual risk and familial liability for suicide attempt and suicide in autism: a population-based study. Psychol Med.

[12] Rattaz C, Michelon C, Munir K, Baghdadli A (2018). Challenging behaviours at early adulthood in autism spectrum disorders: topography, risk factors and evolution. J Intellect Disabil Res.

[13] Rattaz C, Michelon C, Baghdadli A (2015). Symptom severity as a risk factor for self-injurious behaviours in adolescents with autism spectrum disorders. J Intellect Disabil Res.

[14] Russell KM, Frost KM, Ingersoll B (2019). The relationship between subtypes of repetitive behaviors and anxiety in children with autism spectrum disorder. Res Autism Spectr Disord.

[15] Rojahn J, Barnard-Brak L, Medeiros K, Schroeder S (2016). Stereotyped behaviours as precursors of self-injurious behaviours: a longitudinal study with infants and toddlers at risk for developmental delay. J Intellect Disabil Res.

[16] Boyd BA, Baranek GT, Sideris J, Poe MD, Watson LR, Patten E (2010). Sensory features and repetitive behaviors in children with autism and developmental delays. Autism Res.

[17] Richards C, Moss J, Nelson L, Oliver C (2016). Persistence of self-injurious behaviour in autism spectrum disorder over 3 years: a prospective cohort study of risk markers. J Neurodev Disord.

[18] Richards C, Davies L, Oliver C (2017). Predictors of self-injurious behavior and self-restraint in autism spectrum disorder: towards a hypothesis of impaired behavioral control. J Autism Dev Disord.

[19] Flowers J, Lantz J, Hamlin T, Simeonsson RJ (2020). Associated factors of self-injury among adolescents with autism spectrum disorder in a community and residential treatment setting. J Autism Dev Disord.

[20] Vandewalle K, Melia Y (2021). Psychosocial and behavioural factors associated with self injurious behaviour (SIB) in individuals with autism spectrum disorders (ASD). Res Autism Spectr Disord.

[21] Maddox BB, Trubanova A, White SW (2016). Untended wounds: non-suicidal self-injury in adults with autism spectrum disorder. Autism..

[22] Moseley RL, Gregory NJ, Smith P, Allison C, Baron-Cohen S (2019). A ‘choice’, an ‘addiction’, a way ‘out of the lost’: exploring self-injury in autistic people without intellectual disability. Mol Autism..

[23] Goldfarb Y, Zafrani O, Hedley D, Yaari M, Gal E (2021). Autistic adults’ subjective experiences of hoarding and self-injurious behaviors. Autism.

[24] Hunsche MC, Saqui S, Mirenda P, Zaidman-Zait A, Bennett T, Duku E (2020). Parent-reported rates and clinical correlates of suicidality in children with autism spectrum disorder: a longitudinal study. J Autism Dev Disord.

[25] Steenfeldt-Kristensen C, Jones CA, Richards C (2020). The prevalence of self-injurious behaviour in autism: A meta-analytic study. J Autism Dev Disord.

[26] Oliphant RY, Smith EM, Grahame V (2020). What is the prevalence of self-harming and suicidal behaviour in under 18s with ASD, with or without an intellectual disability?. J Autism Dev Disord.

[27] Akram B, Batool M, Rafi Z, Akram A (2017). Prevalence and predictors of non-suicidal self-injury among children with autism spectrum disorder. Pak J Med Sci.

[28] Licence L, Oliver C, Moss J, Richards C (2020). Prevalence and risk-markers of self-harm in autistic children and adults. J Autism Dev Disord.

[29] Conner CM, Golt J, Righi G, Shaffer R, Siegel M, Mazefsky CA (2020). A comparative study of suicidality and its association with emotion regulation impairment in large ASD and US census-matched samples. J Autism Dev Disord.

[30] Cassidy S, Bradley L, Shaw R, Baron-Cohen S (2018). Risk markers for suicidality in autistic adults. Mol Autism..

[31] Moseley RL, Gregory NJ, Smith P, Allison C, Baron-Cohen S (2020). Links between self-injury and suicidality in autism. Mol Autism.

[32] Joiner TE (2005). Why people die by suicide.

[33] Van Orden KA, Witte TK, Cukrowicz KC, Braithwaite SR, Selby EA, Joiner TE (2010). The interpersonal theory of suicide. Psychol Rev..

[34] Bender TW, Gordon KH, Bresin K, Joiner TE (2011). Impulsivity and suicidality: The mediating role of painful and provocative experiences. J Affect Disord..

[35] Chu C, Buchman-Schmitt JM, Stanley IH, Hom MA, Tucker RP, Hagan CR (2017). The interpersonal theory of suicide: a systematic review and meta-analysis of a decade of cross-national research. Psychol Bull..

[36] Willoughby T, Heffer T, Hamza CA (2015). The link between nonsuicidal self-injury and acquired capability for suicide: a longitudinal study. J Abnorm Psychol.

[37] Klonsky ED, Saffer BY, Bryan CJ (2018). Ideation-to-action theories of suicide: a conceptual and empirical update. Curr Opin Psychol.

[38] Chu C, Hom MA, Stanley IH, Gai AR, Nock MK, Gutierrez PM (2018). Non-suicidal self-injury and suicidal thoughts and behaviors: A study of the explanatory roles of the interpersonal theory variables among military service members and veterans. J Consult Clin Psychol.

[39] Ren Y, You J, Zhang X, Huang J, Conner BT, Sun R (2019). Differentiating suicide attempters from suicide ideators: the role of capability for suicide. Arch Suicide Res.

[40] Mbroh H, Zullo L, Westers N, Stone L, King J, Kennard B (2018). Double trouble: nonsuicidal self-injury and its relationship to suicidal ideation and number of past suicide attempts in clinical adolescents. J Affect Disord.

[41] Ammerman BA, Burke TA, Alloy LB, McCloskey MS (2016). Subjective pain during NSSI as an active agent in suicide risk. Psychiatry Res.

[42] Franklin JC, Puzia ME, Lee KM, Lee GE, Hanna EK, Spring VL (2013). The nature of pain offset relief in nonsuicidal self-injury: a laboratory study. Clin Psychol Sci.

[43] Matney J, Westers NJ, Horton SE, King JD, Eaddy M, Emslie GJ (2018). Frequency and methods of nonsuicidal self-injury in relation to acquired capability for suicide among adolescents. Arch Suicide Res.

[44] George SE, Page AC, Hooke GR, Stritzke WGK (2016). Multifacet assessment of capability for suicide: development and prospective validation of the acquired capability with rehearsal for suicide scale. Psychol Assess.

[45] Knorr AC, Ammerman BA, Hamilton AJ, McCloskey MS (2019). Predicting status along the continuum of suicidal thoughts and behavior among those with a history of nonsuicidal self-injury. Psychiatry Res.

[46] Victor S, Klonsky E (2014). Correlates of suicide attempts among self-injurers: a meta-analysis. Clin Psychol Rev.

[47] Ammerman BA, Jacobucci R, Turner BJ, Dixon-Gordon KL, McCloskey MS (2020). Quantifying the importance of lifetime frequency versus number of methods in conceptualizing nonsuicidal self-injury severity. Psychol Violence.

[48] Gauthier JM, Hollingsworth DW, Bagge CL (2018). Number and violence of suicide attempt methods: a preliminary investigation of the associations with fearlessness of suicide and fearlessness about death. Psychiatry Res.

[49] O’Loughlin CM, Culianos D, Park Y, Serang S, Ammerman BA (2021). Implementing exploratory mediation to clarify the nonsuicidal self-injury–suicidality connection. J Psychopathol Behav Assess.

[50] Ammerman BA, Hong M, Sorgi K, Park Y, Jacobucci R, McCloskey MS (2019). An examination of individual forms of nonsuicidal self-injury. Psychiatry Res.

[51] Baer MM, Tull MT, Forbes CN, Richmond JR, Gratz KL (2020). Methods matter: nonsuicidal self-injury in the form of cutting is uniquely associated with suicide attempt severity in patients with substance use disorders. Suicide Life Threat Behav.

[52] Pelton MK, Crawford H, Robertson AE, Rodgers J, Baron-Cohen S, Cassidy S (2020). Understanding suicide risk in autistic adults: comparing the interpersonal theory of suicide in autistic and non-autistic samples. J Autism Dev Disord..

[53] Dow D, Morgan L, Hooker JL, Michaels MS, Joiner TE, Woods J (2019). Anxiety, Depression, and the interpersonal theory of suicide in a community sample of adults with autism spectrum disorder. Arch Suicide Res..

[54] Moseley R, Gregory NJ, Smith P, Allison C, Cassidy S, Baron-Cohen S (2022). The relevance of the interpersonal theory of suicide for predicting past-year and lifetime suicidality in autistic adults. Mol Autism.

[55] Whitlock J, Exner-Cortens D, Purington A (2014). Assessment of nonsuicidal self-injury: development and initial validation of the Non-Suicidal Self-Injury-Assessment Tool (NSSI-AT). Psychol Assess.

[56] Shahnaz A, Bauer BW, Daruwala SE, Klonsky ED (2020). Exploring the scope and structure of suicide capability. Suicide Life Threat Behav.

[57] Rogers ML, Bauer BW, Gai AR, Duffy ME, Joiner TE (2021). Examination of measurement invariance of the acquired capability for suicide scale. Psychol Assess..

[58] Nock MK, Holmberg EB, Photos VI, Michel BD (2007). Self-injurious thoughts and behaviors interview: development, reliability, and validity in an adolescent sample. Psychol Assess.

[59] Kroenke K, Spitzer RL (2002). The PHQ-9: a new depression diagnostic and severity measure. Psychiatr Ann.

[60] Arnold SR, Uljarević M, Hwang YI, Richdale AL, Trollor JN, Lawson LP (2020). Brief report: psychometric properties of the Patient Health Questionaire-9 (PHQ-9) in Autistic Adults. J Autism Dev Disord.

[61] Manea L, Gilbody S, McMillan D (2012). Optimal cut-off score for diagnosing depression with the Patient Health Questionnaire (PHQ-9): A meta-analysis. CMAJ.

[62] Kane L, Ashbaugh AR (2017). Simple and parallel mediation: a tutorial exploring anxiety sensitivity, sensation seeking, and gender. Quant Methods Psychol.

[63] Sara G (2010). What's normal anyway? Normal and non-normal distributions in psychiatry. Acta Neuropsychiatrica.

[64] Hayes AF (2017). An introduction to mediation, moderation, and conditional process analysis: a regression-based approach.

[65] Koenig J, Thayer J, Kaess M (2016). A meta-analysis on pain sensitivity in self-injury. Psychol Med.

[66] Kirtley OJ, O’Carroll RE, O’Connor RC (2016). Pain and self-harm: a systematic review. J Affect Disord.

[67] Griffiths S, Allison C, Kenny R, Holt R, Smith P, Baron-Cohen S (2019). The vulnerability experiences quotient (VEQ): a study of vulnerability, mental health and life satisfaction in autistic adults. Autism Res.

[68] Hoover DW, Kaufman J (2018). Adverse childhood experiences in children with autism spectrum disorder. Curr Opin Psychiatry.

[69] Moseley R, Turner-Cobb J, Spahr CM, Shields GS, Slavich GM (2021). Lifetime and perceived stress, social support, loneliness, and health in autistic adults. Health Psychol.

[70] Spangenberg L, Glaesmer H, Hallensleben N, Rath D, Forkmann T (2019). (In)stability of capability for suicide in psychiatric inpatients: longitudinal assessment using ecological momentary assessments. Suicide Life Threat Behav.

[71] Preece D, Kiekens G, Boyes M, Mortier P, Nock M, Kessler R (2021). Acquired capability for suicide among Belgian and Australian University students: psychometric properties of the German capability for suicide questionnaire and a test of the interpersonal theory of suicide. Suicide Life Threat Behav.

[72] van der Venne P, Balint A, Drews E, Parzer P, Resch F, Koenig J (2021). Pain sensitivity and plasma beta-endorphin in adolescent non-suicidal self-injury. J Affect Disord.

[73] Selby EA, Kranzler A, Lindqvist J, Fehling KB, Brillante J, Yuan F (2019). The dynamics of pain during nonsuicidal self-injury. Clin Psychol Sci.

[74] Harris LM, Ribeiro JD (2021). Does fearlessness about death mediate the association between NSSI and suicide attempts? A longitudinal study of over 1,000 high-risk individuals. J Consult Clin Psychol.

[75] O'Connor RC, Kirtley OJ (2018). The integrated motivational-volitional model of suicidal behaviour. PhilosTrans R Soc B: Biol Sci.

[76] Bryan CJ, Butner JE, May AM, Rugo KF, Harris JA, Oakey DN (2020). Nonlinear change processes and the emergence of suicidal behavior: A conceptual model based on the fluid vulnerability theory of suicide. New Ideas Psychol.

[77] Schuler KR, Smith PN, Rufino KA, Stuart GL, Wolford-Clevenger C (2020). Examining the temporal stability of suicide capability among undergraduates: a latent growth analysis. J Affect Disord.

[78] Klonsky DE, May AM (2015). The three-step theory (3ST): a new theory of suicide rooted in the "ideation-to-action" framework. Int J Cogn Ther.

[79] Anestis MD, Law KC, Jin H, Houtsma C, Khazem LR, Assavedo BL (2017). Treating the capability for suicide: a vital and understudied frontier in suicide prevention. Suicide Life Threat Behav.

[80] Branley-Bell D, O'Connor DB, Green JA, Ferguson E, O'Carroll RE, O'Connor RC (2019). Distinguishing suicide ideation from suicide attempts: further test of the integrated motivational-volitional model of suicidal behaviour. J Psychiatr Res.

[81] Ribeiro JD, Linthicum KP, Joiner TE, Huang X, Harris LM, Bryen CP (2021). Do suicidal desire and facets of capability for suicide predict future suicidal behavior? A longitudinal test of the desire–capability hypothesis. J Abnorm Psychol.

[82] Assavedo BL, Anestis MD (2016). The relationship between non-suicidal self-injury and both perceived burdensomeness and thwarted belongingness. J Psychopathol Behav Assess.

[83] Chu C, Rogers ML, Joiner TE (2016). Cross-sectional and temporal association between non-suicidal self-injury and suicidal ideation in young adults: the explanatory roles of thwarted belongingness and perceived burdensomeness. Psychiatr Res.

[84] Ammerman BA, Sorgi KM, Fahlgren MK, Puhalla AA, McCloskey MS (2021). An experimental examination of interpersonal problem-solving in nonsuicidal self-injury: a pilot study. J Psychiatr Res.

[85] Victor SE, Scott LN, Stepp SD, Goldstein TR (2019). I want you to want me: Interpersonal stress and affective experiences as within-person predictors of nonsuicidal self-injury and suicide urges in daily life. Suicide Life Threat Behav.

[86] Zhang X, Ren Y, You J, Huang C, Jiang Y, Lin M-P (2017). Distinguishing pathways from negative emotions to suicide ideation and to suicide attempt: The differential mediating effects of nonsuicidal self-injury. J Abnorm Child Psychol.

[87] Davis KC, Anderson JL (2021). Psychological pain: a moderating factor between personality psychopathology and self-harm. J Am Coll Health..

[88] Rizvi SJ, Iskric A, Calati R, Courtet P (2017). Psychological and physical pain as predictors of suicide risk: evidence from clinical and neuroimaging findings. Curr Opin Psychiatry.

[89] Hedley D, Uljarević M, Cai RY, Bury SM, Stokes MA, Evans DW (2021). Domains of the autism phenotype, cognitive control, and rumination as transdiagnostic predictors of DSM-5 suicide risk. PLoS ONE.

[90] South M, Beck J, Lundwall R, Christensen M, Cutrer E, Gabrielsen T (2020). Unrelenting depression and suicidality in women with autistic traits. J Autism Dev Disord.

[91] Dell'Osso L, Carpita B, Cremone IM, Muti D, Diadema E, Barberi FM (2019). The mediating effect of trauma and stressor related symptoms and ruminations on the relationship between autistic traits and mood spectrum. Psychiatry Res.

[92] Costa AP, Loor C, Steffgen G (2020). Suicidality in adults with autism spectrum disorder: the role of depressive symptomatology, alexithymia, and antidepressants. J Autism Dev Disord.

[93] Batterham PJ, Calear AL (2020). Incorporating psychopathology into the interpersonal-psychological theory of suicidal behavior (IPTS). Suicide Life Threat Behav.

[94] Wang S, Li C, Jia X, Lyu J, Wang Y, Sun H (2020). From depressive symptoms to suicide risk: roles of sense of belongingness and acquired capability for suicide in patients with mental disorders. PsyCh J.

[95] Yasuda Y, Hashimoto R, Nakae A, Kang H, Ohi K, Yamamori H (2016). Sensory cognitive abnormalities of pain in autism spectrum disorder: a case-control study. Ann Gen Psychiatry.

[96] Thaler H, Skewes JC, Gebauer L, Christensen P, Prkachin KM, Jegindø Elmholdt E-M (2018). Typical pain experience but underestimation of others’ pain: emotion perception in self and others in autism spectrum disorder. Autism.

[97] Williams ZJ, Failla MD, Davis SL, Heflin BH, Okitondo CD, Moore DJ (2019). Thermal perceptual thresholds are typical in autism spectrum disorder but strongly related to intra-individual response variability. Sci Rep.

[98] Huang X, Ribeiro JD, Franklin JC (2020). The differences between individuals engaging in nonsuicidal self-injury and suicide attempt are complex (vs. complicated or simple). Front Psychiatry.

[99] Wester KL, McKibben WB (2016). Participants' experiences of non-suicidal self-injury: supporting existing theory and emerging conceptual pathways. J Mental Health Couns.

[100] Glenn CR, Klonsky ED (2010). The role of seeing blood in non-suicidal self-injury. J Clin Psychol.

[101] Hasking PA, Di Simplicio M, McEvoy PM, Rees CS (2018). Emotional cascade theory and non-suicidal self-injury: the importance of imagery and positive affect. Cogn Emot.

[102] Brackman EH, Morris BW, Andover MS (2016). Predicting risk for suicide: a preliminary examination of non-suicidal self-injury and the acquired capability construct in a college sample. Arch Suicide Res.

[103] Hamza CA, Stewart SL, Willoughby T (2012). Examining the link between nonsuicidal self-injury and suicidal behavior: a review of the literature and an integrated model. Clin Psychol Rev.

[104] Peterson AL, Chen JI, Karver MS, Labouliere CD (2019). Frustration with feeling: Latent classes of non-suicidal self-injury and emotion regulation difficulties. Psychiatry Res.

[105] Anestis MD, Khazem LR, Law KC (2015). How many times and how many ways: The impact of number of nonsuicidal self-injury methods on the relationship between nonsuicidal self-injury frequency and suicidal behavior. Suicide Life Threat Behav..

[106] Bracken-Minor KL, McDevitt-Murphy ME (2014). Differences in features of non-suicidal self-injury according to borderline personality disorder screening status. Arch Suicide Res.

[107] Somer O, Bildik T, Kabukçu-Başay B, Güngör D, Başay Ö, Farmer RF (2015). Prevalence of non-suicidal self-injury and distinct groups of self-injurers in a community sample of adolescents. Soc Psychiatry Psychiatr Epidemiol.

[108] Klonsky ED, Olino TM (2008). Identifying clinically distinct subgroups of self-injurers among young adults: a latent class analysis. J Consult Clin Psychol.

[109] Whitlock J, Muehlenkamp J, Eckenrode J (2008). Variation in nonsuicidal self-injury: Identification and features of latent classes in a college population of emerging adults. J Clin Child Adolesc Psychol.

[110] Huang X, Ribeiro JD, Franklin JC (2020). The differences between suicide ideators and suicide attempters: Simple, complicated, or complex?. J Consult Clin Psychol.

[111] Joiner TE, Jeon ME, Lieberman A, Janakiraman R, Duffy ME, Gai AR (2021). On prediction, refutation, and explanatory reach: a consideration of the interpersonal theory of suicidal behavior. Prev Med.

[112] Kleiman EM, Nock MK (2018). Real-time assessment of suicidal thoughts and behaviors. Curr Opin Psychol.

[113] Turner BJ, Layden BK, Butler SM, Chapman AL (2013). How often, or how many ways: clarifying the relationship between non-suicidal self-injury and suicidality. Arch Suicide Res..

[114] Stanley IH, Hom MA, Rogers ML, Hagan CR, Joiner TE (2016). Understanding suicide among older adults: a review of psychological and sociological theories of suicide. Aging Ment Health.

[115] Batterham PJ, Walker J, Leach LS, Ma J, Calear AL, Christensen H (2018). A longitudinal test of the predictions of the interpersonal-psychological theory of suicidal behaviour for passive and active suicidal ideation in a large community-based cohort. J Affect Disord.

[116] Hafford-Letchfield T, Jain B, Gleeson H, Roesch C, Ellmers T (2022). A scoping review exploring the ‘grey area’of suicide-related expression in later life: developing a conceptual framework for professional engagement. Ageing Soc..

[117] Whitlock J, Selekman M, Nock MK (2014). Nonsuicidal self-injury across the life span. The Oxford handbook of suicide and self-injury.

[118] Peterson RA, Merunka DR (2014). Convenience samples of college students and research reproducibility. J Bus Res.

[119] Mandy W, Midouhas E, Hosozawa M, Cable N, Sacker A, Flouri E (2022). Mental health and social difficulties of late-diagnosed autistic children, across childhood and adolescence. J Child Psychol Psychiatry..

[120] Hull L, Petrides KV, Mandy W (2020). Cognitive predictors of self-reported camouflaging in autistic adolescents. Autism Res.

[121] Livingston LA, Colvert E, Bolton P, Happé F (2019). Good social skills despite poor theory of mind: exploring compensation in autism spectrum disorder. J Child Psychol Psychiatry.

[122] Abbott P, Happé FG, Charlton RA (2018). Exploratory study of executive function abilities across the adult lifespan in individuals receiving an ASD diagnosis in adulthood. J Autism Dev Disord.

[123] Rødgaard EM, Jensen K, Miskowiak KW, Mottron L (2022). Representativeness of autistic samples in studies recruiting through social media. Autism Res.

[124] Rubenstein E, Furnier S (2021). # Bias: The opportunities and challenges of surveys that recruit and collect data of autistic adults online. Autism Adulthood.

[125] Huang Y, Arnold SR, Foley KR, Lawson LP, Richdale AL, Trollor JN (2021). Factors associated with age at autism diagnosis in a community sample of Australian adults. Autism Res.

[126] Jadav N, Bal VH (2022). Associations between co-occurring conditions and age of autism diagnosis: Implications for mental health training and adult autism research. Autism Res..

[127] Haas K, Costley D, Falkmer T, Richdale A, Sofronoff K. Optimising the recruitment and retention of adults for longitudinal autism spectrum research: a mixed methods study. Full report. Cooperative Research Centre for Living with Autism Spectrum Disorders, Brisbane. Copies of this report can be downloaded from the Autism CRC website 2014. www.autismcrc.com.au.

