# Supplementary material for: Non-suicidal self-injury and its relation to suicide through acquired capability: investigating this causal mechanism in a mainly late-diagnosed autistic sample
Source: Mol Autism. 2022 Nov 12;13:45. doi: 10.1186/s13229-022-00522-5 (PMC9655904; doi:10.1186/s13229-022-00522-5)
Supplement: Supplementary file 1 — Additional file 1. Supplementary data analysis. [file 13229_2022_522_MOESM1_ESM.docx]

**Supplementary materials**

Supplementary item 1: Full statistical notations for moderated-mediation models

Supplementary item 2: Full statistical notations for sequential mediation with scratching

Supplementary item 3: Full statistical notations for sequential mediation with cutting

Supplementary item 4: Full statistical notations for sequential mediation with self-hitting

Supplementary item 5: Full statistical notations for sequential mediation with range of NSSI

**Supplementary item 1: Full statistical notations for moderated-mediation models**

In order to streamline the manuscript and because they were not our focus, we did not describe all effects of covariates for the analysis presented in Part 1 of the Results section, which can be seen here in their entirety. Confidence intervals (95%) are presented for significant effects.

|  | **Relationship to pain tolerance (path A^1^)** | | | | | **Relationship to reduced fear of death by suicide (path A^2^)** | | | | | **Relationship to mental rehearsal of suicide plans (path A^3^)** | | | | |
| --- | --- | --- | --- | --- | --- | --- | --- | --- | --- | --- | --- | --- | --- | --- | --- |
|  | ***B (SE)*** | ***t*** | | ***p (CI)*** | | ***B (SE)*** | | ***t*** | ***p (CI)*** | | ***B (SE)*** | | ***t*** | | ***p (CI)*** |
| Lifetime frequency of NSSI | .48 (.11) | 4.41 | | < .001  (.27, .70) | | .46 (.11) | | 4.22 | < .001  (.25, .68) | | 1.29 (.15) | | | 8.38 | < .001  (.99, 1.60) |
| Diagnosis (main effect of moderator) | -.37 (.72) | -.52 | | .6061 | | 1.55 (.71) | | 2.18 | .0299  (.15, 2.95) | | 6.37 (1.00) | | | 6.35 | < .001  (4.40, 8.34) |
| Interaction (moderation effect of Diagnosis) | -.14 (.14) | -1.02 | | .3101 | | -.24 (.14) | | -1.77 | .0767 | | -.83 (.19) | | | -4.26 | < .001  (-1.20, -.44) |
|  |  |  | |  | |  | |  |  | | Autistic: B = .47 (SE: .13), t = 3.58, p = .0004, CI: .21, .73  Non-autistic: B = 1.29 (SE: .15), t = 8.38, p < .001, CI: .99, 1.60) | | | | |
| Age (covariate) | .01 (.02) | .42 | | .6749 | | .05 (.02) | | 2.71 | .0069  (.01, 09) | | -.03 (.03) | | | -1.09 | .2774 |
| Sex (covariate) | -.08 (.50) | -.16 | | .8731 | | .88 (.50) | | 1.77 | .0778 | | 1.12 (.70) | | | 1.59 | .1116 |
| PHQ-9 scores (covariate) | .12 (.03) | 4.26 | | < .001  (.07, .18) | | .10 (.03) | | 3.30 | .0010  (.04, .15) | | .44 (.04) | | | 10.63 | < .001  (.35, .52) |
| Model | *R^2^* = .12, *F* (6, 619) = 14.13, *p* < .001 | | | | | *R^2^* = .17, *F* (6, 619) 21.05, *p* < .001 | | | | | *R^2^* = .44, *F* (6, 619) 81.66, *p* < .001 | | | | |
| **Relationship to lifetime suicide attempts** | | | | | | | | | | | | | | | |
|  | | | | | ***B (SE)*** | | ***t*** | | | ***p (CI)*** | | | | | |
| Lifetime frequency of NSSI (direct effect: path c’) | | | | | .10 (.03) | | 3.25 | | | .0012 (.04, .15) | | | | | |
| Pain tolerance (path B^1^) | | | | | .01 (.01) | | 1.04 | | | .2978 | | | | | |
| Reduced fear of death by suicide (path B^2^) | | | | | .04 (.02) | | 2.70 | | | .0072 (.01, .07) | | | | | |
| Mental rehearsal of suicide plans (path B^3^) | | | | | .03 (.01) | | 2.68 | | | .0076 (.01, .05) | | | | | |
| Diagnosis (main effect) | | | | | .14 (.21) | | .68 | | | .4986 | | | | | |
| Interaction 1 (path B^1^) | | | | | -.00 (.04) | | -.03 | | | .9723 | | | | | |
| Interaction 2 (path B^2^) | | | | | .00 (.02) | | .11 | | | .9086 | | | | | |
| Interaction 3 (path B^3^) | | | | | .02 (.02) | | 1.13 | | | .2594 | | | | | |
| Interaction 4 (direct effect) | | | | | -.00 (.01) | | -.34 | | | .7306 | | | | | |
| Age (covariate) | | | | | -.00 (.00) | | -1.14 | | | .2546 | | | | | |
| Sex (covariate) | | | | | -.24 (.11) | | -2.07 | | | .0393 (-.46, -.01) | | | | | |
| PHQ-9 scores (covariate) | | | | | .02 (.01) | | 2.83 | | | .0049 (.01, .03) | | | | | |
| **Model** | | | | | *R^2^* = .34, *F* (12, 613) = 26.63, *p* < .001 | | | | | | | | | | |
| **Direct and indirect effects** | | | | | | | | | | | | | | | |
|  | | | ***B (SE)*** | | | | ***t*** | | | | | ***p (CI)***  ***/ bootstrapped CI*** | | | |
| Direct effect of NSSI on outcome  variable  Autistic  Non-autistic | | | .09 (.03)  .10 (.03) | | | | 4.30  3.25 | | | | | < .001 (.05, .14)  .0012 (.04, .15) | | | |
| Indirect effect of NSSI via pain  tolerance  Autistic  Non-autistic | | | .01 (.01)  .01 (.01) | | | |  | | | | | -.004, .02  -.01, .02 | | | |
|  | | | Index of moderated mediation: NS  (B = .-.00 (.01), bootCI: -.02, .02) | | | | | | | | | | | | |
| Indirect effect of NSSI via reduced  fear of death  Autistic  Non-autistic | | | .01 (.01)  .02 (.01) | | | |  | | | | | .0007, .03  .01, .03 | | | |
|  | | | Index of moderated mediation: NS  (B = .-.01 (.01), bootCI: -.03, .02) | | | | | | | | | | | | |
| Indirect effect of NSSI via mental  rehearsal of suicide  Autistic  Non-autistic | | | .01 (.01)  .03 (.01) | | | |  | | | | | .0007, .02  .02, .05 | | | |
|  | | | Index of moderated mediation: B = .-.02 (.01), bootCI: -.05, -.00) | | | | | | | | | | | | |

**Supplementary item 2: Full statistical notations for sequential mediation with scratching**

The below sequential mediation analysis (Model 6) was run for autistic participants (presented in blue) and non-autistic participants (orange) separately. Note: these are presented together for visual comparison, but **were not statistically compared**.

|  | **Relationship to average pain inflicted during NSSI (mediator 1)** | | | | **Relationship to habituation to NSSI (mediator 2)** | | | | **Relationship to acquired capability (mediator 3)** | | | | | |
| --- | --- | --- | --- | --- | --- | --- | --- | --- | --- | --- | --- | --- | --- | --- |
|  | ***B (SE)*** | ***t*** | | ***p (CI)*** | ***B (SE)*** | ***t*** | | ***p (CI)*** | ***B (SE)*** | | ***t*** | | | ***p (CI)*** |
| Scratching (predictor) | .49 (.33)  -.03 (.34) | 1.45  -.10 | | .1481  .9244 | .21 (.44)  .56 (.41) | .48  1.36 | | .6329  .1762 | -1.53 (1.79)  .77 (1.87) | | | -.85  .41 | | .3954  .6807 |
| PHQ-9 scores (covariate) | -.01 (.02)  .04 (.02) | -.27  1.50 | | .7876  .1362 | .18 (.03)  .11 (.03) | 6.51  4.08 | | < .001  .0001 | .51 (.12)  1.01 (.13) | | | 4.23  7.53 | | < .001  < .001 |
| Average pain inflicted during NSSI (mediator 1) |  |  | |  | .07 (.09)  -.06 (.10) | .80  -.61 | | .4245  .5429 | -.07 (.36)  -.27 (.44) | | | -.19  -.62 | | .8514  .5377 |
| Habituation to NSSI (mediator 2) |  |  | |  |  |  | |  | .83 (.28)  1.45 (.37) | | | 2.99  3.93 | | .0032  .0001 |
| Model | *R^2^* = .01, *F* (2, 217) = 1.07, *p* = .3456  *R^2^* = .01, *F* (2, 153) = 1.13, *p* = .3258 | | | | *R^2^* = .17, *F* (3, 216) = 14.64, *p* < .001  *R^2^* = .12, *F* (3, 152) = 6.84, *p* = .0002 | | | | *R^2^* = .20, *F* (4, 215) = 11.11, *p* < .001  *R^2^* = .41, *F* (4, 151) = 26.44, *p* < .001 | | | | | |
|  | **Relationship to lifetime suicide attempts** | | | | | | **Direct and indirect effects** | | | | | | | |
|  | ***B (SE)*** | | ***t*** | | ***p (CI)*** | |  | | | ***B (SE)*** | | | ***Bootstrapped CI*** | |
| Scratching (predictor: direct effect or path c’) | .05 (.21)  -.19 (.19) | | .21  -1.02 | | .8271  .3077 | | Indirect effect of scratching via average pain inflicted during NSSI (mediator 1) | | | .00 (.05)  -.00 (.02) | | | -.06, .05  -.04, .03 | |
| PHQ-9 scores (covariate) | .01 (.01)  .04 (.02) | | 1.01  2.39 | | .3131  .0181 | | Indirect effect of scratching via habituation (mediator 2) | | | .01 (.02)  .03 (.04) | | | -.04, .07  -.04, .11 | |
| Average pain inflicted during NSSI (mediator 1) | .00 (.04)  .02 (.04) | | .01  .44 | | .9898  .6610 | | Indirect effect of scratching via acquired capability (mediator 3) | | | -.06 (.07)  .03 (.07) | | | -.20, .07  -.11, .16 | |
| Habituation to NSSI (mediator 2) | .03 (.03)  .05 (.04) | | .98  1.30 | | .3268  .1957 | | Indirect effect of scratching via mediators 1 and 2 | | | .00 (.00)  .00 (.00) | | | -.005, .01  -.005, .01 | |
| Acquired capability (mediator 3) | .04 (.01)  .04 (.01) | | 4.53  4.54 | | < .001  < .001 | | Indirect effect of scratching via mediators 1 and 3 | | | -.00 (.01)  .00 (.03) | | | -.02, .01  -.01, .02 | |
| Model | *R^2^* = .14, *F* (5, 214) = 7.22, *p* < .001  *R^2^* = .34, *F* (5, 150) = 15.20, *p* < .001 | | | | | | Indirect effect of scratching via mediators 2 and 3 | | | .01 (.01)  .03 (.03) | | | -.02, .04  -.02, .09 | |
|  |  |  |  |  |  |  | Indirect effect of scratching via mediators 1, 2 and 3 | | | .00 (.00)  .00 (.00) | | | -.003, .01  -.004, .00 | |

**Supplementary item 3: Full statistical notations for sequential mediation with cutting**

The below sequential mediation analysis (Model 6) was run for autistic participants (presented in blue) and non-autistic participants (orange) separately. Note: these are presented together for visual comparison, but **were not statistically compared**.

|  | **Relationship to average pain inflicted during NSSI (mediator 1)** | | | | **Relationship to habituation to NSSI (mediator 2)** | | | | **Relationship to acquired capability (mediator 3)** | | | | | |
| --- | --- | --- | --- | --- | --- | --- | --- | --- | --- | --- | --- | --- | --- | --- |
|  | ***B (SE)*** | ***t*** | | ***p (CI)*** | ***B (SE)*** | ***t*** | | ***p (CI)*** | ***B (SE)*** | | ***t*** | | | ***p (CI)*** |
| Cutting (predictor) | -.18 (.31)  .78 (.32) | -.57  2.47 | | .5668  .0146 | .98 (.39)  1.07 (.38) | 2.50  2.79 | | .0131  .0060 | 5.68 (1.60)  2.90 (1.81) | | | 3.54  1.61 | | .0005  .1097 |
| PHQ-9 scores (covariate) | -.00 (.02)  .02 (.02) | -.07  1.04 | | .9427  .2992 | .17 (.09)  .11 (.03) | 6.14  3.88 | | < .001  .0002 | .47 (.12)  1.00 (.13) | | | 3.99  7.50 | | .0001  < .001 |
| Average pain inflicted during NSSI (mediator 1) |  |  | |  | .08 (.09)  -.11 (.10) | .95  -1.17 | | .3431  .2453 | -.04 (.35)  -.42 (.45) | | | -.10  -.95 | | .9175  .3441 |
| Habituation to NSSI (mediator 2) |  |  | |  |  |  | |  | .66 (.27)  1.33 (.37) | | | 2.41  3.58 | | .0167  .0005 |
| Model | *R^2^* = .00, *F* (2, 218) = .18, *p* = .8362  *R^2^* = .05, *F* (2, 153) = 4.22, *p* = .0164 | | | | *R^2^* = .19, *F* (3, 217) = 17.03, *p* < .001  *R^2^* = .15, *F* (3, 152) = 9.06, *p* < .001 | | | | *R^2^* = .21, *F* (4, 216) = 14.72, *p* < .001  *R^2^* = .42, *F* (4, 151) = 27.47, *p* < .001 | | | | | |
|  | **Relationship to lifetime suicide attempts** | | | | | | **Direct and indirect effects** | | | | | | | |
|  | ***B (SE)*** | | ***t*** | | ***p (CI)*** | |  | | | ***B (SE)*** | | | ***Bootstrapped CI*** | |
| Cutting (predictor: direct effect or path c’) | .96 (.19)  .60 (.18) | | 5.16  3.36 | | < .001  .0010 | | Indirect effect of cutting via average pain inflicted during NSSI (mediator 1) | | | -.00 (.01)  -.01 (.04) | | | -.03, .03  -.09, .08 | |
| PHQ-9 scores (covariate) | .01 (.01)  .04 (.02) | | .98  2.38 | | .3267  .0187 | | Indirect effect of cutting via habituation (mediator 2) | | | .01 (.03)  .03 (.05) | | | -.05, .09  -.07, .14 | |
| Average pain inflicted during NSSI (mediator 1) | .01 (.04)  -.01 (.04) | | .27  -.29 | | .7866  .7709 | | Indirect effect of cutting via acquired capability (mediator 3) | | | **.15 (.07)**  **.10 (.07)** | | | **.04, .30**  **-.02, .24** | |
| Habituation to NSSI (mediator 2) | .01 (.03)  .02 (.04) | | .43  .65 | | .6665  .5194 | | Indirect effect of cutting via mediators 1 and 2 | | | -.00 (.00)  -.00 (.01) | | | -.004, .00  -.02, .01 | |
| Acquired capability (mediator 3) | .03 (.01)  .03 (.01) | | 3.47  4.18 | | .0006  < .001 | | Indirect effect of cutting via mediators 1 and 3 | | | .00 (.00)  -.01 (.01) | | | -.01, .01  -.04, .01 | |
| Model | *R^2^* = .24, *F* (5, 215) = 13.48, *p* < .001  *R^2^* = .38, *F* (5, 150) = 18.27, *p* < .001 | | | | | | Indirect effect of cutting via mediators 2 and 3 | | | **.02 (.01)**  **.05 (.02)** | | | **.0007, .04**  **.01, .11** | |
|  |  |  |  |  |  |  | Indirect effect of cutting via mediators 1, 2 and 3 | | | -.00 (.00)  -.00 (.00) | | | -.003, .00  -.01, .00 | |

**Supplementary item 4: Full statistical notations for sequential mediation with self-hitting**

The below sequential mediation analysis (Model 6) was run for autistic participants (presented in blue) and non-autistic participants (orange) separately. Note: these are presented together for visual comparison, but **were not statistically compared**.

|  | **Relationship to average pain inflicted during NSSI (mediator 1)** | | | | **Relationship to habituation to NSSI (mediator 2)** | | | | **Relationship to acquired capability (mediator 3)** | | | | | |
| --- | --- | --- | --- | --- | --- | --- | --- | --- | --- | --- | --- | --- | --- | --- |
|  | ***B (SE)*** | ***t*** | | ***p (CI)*** | ***B (SE)*** | ***t*** | | ***p (CI)*** | ***B (SE)*** | | ***t*** | | | ***p (CI)*** |
| Self-hitting (predictor) | .49 (.30)  .70 (.34) | 1.63  2.04 | | .1046  .0426 | 1.07 (.38)  .83 (.42) | 2.78  2.00 | | .0059  .0471 | 4.77 (1.60)  5.37 (1.87) | | | 2.99  2.87 | | .0031  .0047 |
| PHQ-9 scores (covariate) | -.01 (.02)  .02 (.02) | -.26  .72 | | .7975  .4721 | .17 (.09)  .10 (.03) | 6.51  3.44 | | < .001  .0007 | .52 (.12)  .91 (.14) | | | 4.36  6.74 | | < .001  < .001 |
| Average pain inflicted during NSSI (mediator 1) |  |  | |  | .05 (.09)  -.09 (.10) | .55  -.94 | | .5853  .3473 | -.20 (.36)  -.49 (.43) | | | -.58  -1.12 | | .5657  .2642 |
| Habituation to NSSI (mediator 2) |  |  | |  |  |  | |  | .67 (.28)  1.30 (.36) | | | 2.42  3.60 | | .0164  .0004 |
| Model | *R^2^* = .01, *F* (2, 218) = .15, *p* = .2633  *R^2^* = .04, *F* (2, 153) = 3.25, *p* = .0416 | | | | *R^2^* = .20, *F* (3, 217) = 17.61, *p* < .001  *R^2^* = .13, *F* (3, 152) = 7.65, *p* < .001 | | | | *R^2^* = .20, *F* (4, 216) = 13.62, *p* < .001  *R^2^* = .44, *F* (4, 151) = 29.86, *p* < .001 | | | | | |
|  | **Relationship to lifetime suicide attempts** | | | | | | **Direct and indirect effects** | | | | | | | |
|  | ***B (SE)*** | | ***t*** | | ***p (CI)*** | |  | | | ***B (SE)*** | | | ***Bootstrapped CI*** | |
| Self-hitting (predictor: direct effect or path c’) | -.09 (.19)  .30 (.20) | | -.47  1.49 | | .6371  .1378 | | Indirect effect of self-hitting via average pain inflicted during NSSI (mediator 1) | | | .00 (.02)  .00 (.04) | | | -.05, .05  -.08, .08 | |
| PHQ-9 scores (covariate) | .01 (.01)  .03 (.02) | | .98  2.13 | | .3259  .0346 | | Indirect effect of self-hitting via habituation (mediator 2) | | | .04 (.04)  .03 (.04) | | | -.03, .13  -.05, .13 | |
| Average pain inflicted during NSSI (mediator 1) | .00 (.04)  .01 (.05) | | .08  .15 | | .9346  .8786 | | Indirect effect of self-hitting via acquired capability (mediator 3) | | | **.18 (.07)**  **.18 (.08)** | | | **.05, .32**  **.05, .36** | |
| Habituation to NSSI (mediator 2) | .04 (.03)  .04 (.04) | | 1.07  1.08 | | .2857  .2815 | | Indirect effect of self-hitting via mediators 1 and 2 | | | .00 (.00)  -.00 (.01) | | | -.004, .01  -.02, .01 | |
| Acquired capability (mediator 3) | .04 (.01)  .03 (.01) | | 4.54  4.07 | | < .001  < .001 | | Indirect effect of self-hitting via mediators 1 and 3 | | | -.00 (.01)  -.01 (.01) | | | -.02, .01  -.04, .01 | |
| Model | *R^2^* = .15, *F* (5, 215) = 7.31, *p* < .001  *R^2^* = .34, *F* (5, 150) = 15.55, *p* < .001 | | | | | | Indirect effect of self-hitting via mediators 2 and 3 | | | **.03 (.02)**  **.04 (.02)** | | | **.002, .06**  **.001, .09** | |
|  |  |  |  |  |  |  | Indirect effect of self-hitting via mediators 1, 2 and 3 | | | .00 (.00)  -.00 (.00) | | | -.002, .00  -.01, .00 | |

**Supplementary item 5: Full statistical notations for sequential mediation with range of NSSI**

The below sequential mediation analysis (Model 6) was run for autistic participants (presented in blue) and non-autistic participants (orange) separately. Note: these are presented together for visual comparison, but **were not statistically compared**.

|  | **Relationship to average pain inflicted during NSSI (mediator 1)** | | | | **Relationship to habituation to NSSI (mediator 2)** | | | | **Relationship to acquired capability (mediator 3)** | | | | | |
| --- | --- | --- | --- | --- | --- | --- | --- | --- | --- | --- | --- | --- | --- | --- |
|  | ***B (SE)*** | ***t*** | | ***p (CI)*** | ***B (SE)*** | ***t*** | | ***p (CI)*** | ***B (SE)*** | | ***t*** | | | ***p (CI)*** |
| Range of NSSI (predictor) | .11 (.06)  .14 (.07) | 1.85  1.89 | | .0652  .0607 | .31 (.08)  .47 (.08) | 4.03  5.73 | | .0001  < .001 | 1.46 (.33)  1.78 (.43) | | | 4.48  4.18 | | < .001  < .001 |
| PHQ-9 scores (covariate) | -.01 (.02)  .01 (.03) | -.50  .51 | | .6159  .6131 | .15 (.03)  .05 (.03) | 5.51  1.75 | | < .001  .0820 | .42 (.12)  .83 (.13) | | | 3.66  6.18 | | .0003  < .001 |
| Average pain inflicted during NSSI (mediator 1) |  |  | |  | .04 (.08)  -.14 (.09) | .42  -1.54 | | .6735  .1265 | -.27 (.34)  -.60 (.42) | | | -.78  -1.43 | | .4368  .1558 |
| Habituation to NSSI (mediator 2) |  |  | |  |  |  | |  | .50 (.28)  .79 (.38) | | | 1.82  2.07 | | .0704  .0398 |
| Model | *R^2^* = .02, *F* (2, 219) = 1.72, *p* = .1820  *R^2^* = .04, *F* (2, 153) = 2.94, *p* = .0516 | | | | *R^2^* = .23, *F* (3, 218) = 21.52, *p* < .001  *R^2^* = .27, *F* (3, 152) = 18.40, *p* < .001 | | | | *R^2^* = .24, *F* (4, 217) = 17.19, *p* < .001  *R^2^* = .47, *F* (4, 151) = 33.77, *p* < .001 | | | | | |
|  | **Relationship to lifetime suicide attempts** | | | | | | **Direct and indirect effects** | | | | | | | |
|  | ***B (SE)*** | | ***t*** | | ***p (CI)*** | |  | | | ***B (SE)*** | | | ***Bootstrapped CI*** | |
| Range of NSSI (predictor: direct effect or path c’) | .19 (.04)  .17 (.05) | | 4.82  3.61 | | < .001  .0004 | | Indirect effect of range of NSSI via average pain inflicted during NSSI (mediator 1) | | | -.00 (.01)  -.00 (.01) | | | -.02, .01  -.02, .01 | |
| PHQ-9 scores (covariate) | .01 (.01)  .03 (.02) | | .76  1.89 | | .4472  .0601 | | Indirect effect of range of NSSI via habituation (mediator 2) | | | .00 (.01)  -.00 (.02) | | | -.02, .02  -.04, .05 | |
| Average pain inflicted during NSSI (mediator 1) | -.02 (.04)  -.01 (.04) | | -.49  -.33 | | .6242  .7456 | | Indirect effect of range of NSSI via acquired capability (mediator 3) | | | **.04 (.01)**  **.05 (.02)** | | | **.01, .07**  **.02, .09** | |
| Habituation to NSSI (mediator 2) | .00 (.03)  -.00 (.04) | | .03  -.04 | | .9800  .9649 | | Indirect effect of range of NSSI via mediators 1 and 2 | | | .00 (.00)  .00 (.00) | | | -.001, .00  -.003, .00 | |
| Acquired capability (mediator 3) | .03 (.01)  .03 (.01) | | 3.17  3.27 | | .0017  .0013 | | Indirect effect of range of NSSI via mediators 1 and 3 | | | -.00 (.00)  -.00 (.00) | | | -.004, .00  -.01, .00 | |
| Model | *R^2^* = .23, *F* (5, 216) = 12.87, *p* < .001  *R^2^* = .38, *F* (5, 150) = 18.78, *p* < .001 | | | | | | Indirect effect of range of NSSI via mediators 2 and 3 | | | .00 (.00)  **.01 (.01)** | | | -.001, .01  **.0004, .03** | |
|  |  |  |  |  |  |  | Indirect effect of range of NSSI via mediators 1, 2 and 3 | | | .00 (.00)  -.00 (.00) | | | -.0002, .00  -.002, .00 | |
